# Surface Composition and Preparation Method for Oxygen Nanobubbles for Drug Delivery and Ultrasound Imaging Applications

**DOI:** 10.3390/nano9010048

**Published:** 2019-01-02

**Authors:** Muhammad Saad Khan, Jangsun Hwang, Kyungwoo Lee, Yonghyun Choi, Jaehee Jang, Yejin Kwon, Jong Wook Hong, Jonghoon Choi

**Affiliations:** 1 School of Integrative Engineering, Chung-Ang University, Seoul 06974, Korea; saad.engr@gmail.com (M.S.K.); isnickawesome@gmail.com (J.H.); orztapa@gmail.com (K.L.); dydgus5057@gmail.com (Y.C.); jjaeh95@gmail.com (J.J.); angang1027@gmail.com (Y.K.); 2 Department of Bionano Technology, Hanyang University, Seoul, Korea; 3 Department of Bionano Engineering, Hanyang University, Ansan, Korea

**Keywords:** oxygen nanobubbles, phospholipids, polyethylene glycol, ultrasound imaging

## Abstract

Phospholipids have been widely investigated for the preparation of liposomes, and micro and nanobubbles. They comprise biocompatible and biodegradable molecules and offer simple preparation with a variety of functions in diagnostic and therapeutic applications. Phospholipids require emulsifiers and surfactants to assemble in the form of bubbles. These surfactants determine the size, zeta potential, and other characteristics of particles. Polyethylene glycol (PEG) and its various derivatives have been employed by researchers to synthesize micro and nanobubbles. The stability of phospholipid-shelled nanobubbles has been reported by various researchers owing to the reduction of surface tension by surfactants in the shell. Nanobubbles have been employed to deliver oxygen to tissues and hypoxic cells. In this study, we investigated the effects of different ratios of phospholipids to PEG on the size, distribution, and characterization of oxygen nanobubbles (ONBs). ONBs were synthesized using a sonication technique. We analyzed and compared the sizes, numbers of generated particles, and zeta potentials of different compositions of ONBs using dynamic light scattering and nanoparticle tracking analysis. Then, we employed these oxygen nanobubbles to enhance the cellular microenvironment and cell viability. ONBs were also investigated for ultrasound imaging.

## 1. Introduction

Novel bionanotechnology employing engineered micro and nano structures has been developed for diagnostic and therapeutic applications in the field of healthcare [1]. Micro and nanobubbles are one of those new assemblies already in use in a variety of medical applications, typically as ultrasound contrast agents. Microbubbles are inherently echogenic, owing to a mismatch of acoustic impedance at the liquid/gas interface, which provides an ultrasonic contrast [2,3]. Although large bubbles would generate a greater contrast in ultrasonic imaging, the size of blood capillaries limits the microbubble size to less than 10 µm. Therefore, microbubbles in the range of 1 to 8 µm are preferred for medical usage in ultrasound contrast agents because they have a resonance frequency in the range of 1 to 12 MHz, which is usually employed in commercial applications [2,4,5,6,7,8,9].

Recently, nanobubbles have been investigated for theranostic (or theragnostic) applications, which involve combinations of diagnostic and therapeutic applications. Nanobubbles mostly consist of similar shell/core compositions, in which the shell is often formed of a unilaminar composition of phospholipids, polymers, or proteins, and the core contains a less soluble gas. In contrast to bilayer liposomes of a similar size, nanobubbles have a monolayer shell with a less-soluble gas core, and, therefore, they can be utilized for gas delivery applications [8,10,11,12]. Nanobubbles are of particular interest, owing to their smaller size, biodegradability, higher surface area, longer half-life, higher cellular uptake, and echogenic properties [13]. Owing to their size in the nanometer range, nanobubbles have been investigated for passive targeting in normal tissues and tumors, considering the opportunity to exploit the enhanced permeability and retention effect [5,14,15,16,17,18]. Yin et al. demonstrated that nanobubbles exhibited similar echogenic properties to microbubbles when high ultrasound frequencies were employed, and nanobubbles were retained in tumors for longer periods compared with microbubbles [19].

The stability of the bubbles is governed by the Laplace pressure, represented in the following equation [2,13,20,21]:

$\Delta P=P_{in}-P_{out}=2\frac{\delta }{r}$ where *δ* is the interfacial tension, and r is the radius of the bubble. The Laplace pressure depends on the size of the bubble and the interfacial pressure at the surface. As indicated by the equation, smaller bubbles experience a higher pressure difference, which leads to their rupture. This equation has been discussed for its application in nanosized bubbles by various researchers [13,22,23,24]. Cavalli et al. [13] discussed the role of surfactants in rthe eduction of the value of δ to stabilize the bubbles at nanosize. Less soluble gas in the core can also promote the stability of the bubbles. Experimental evidences and characterizations by various researchers reveal that nanobubbles can be used in diagnostic and applications [12,16,17,19,25,26]. The main role in the stability of the nanobubbles is the composition of shell and surfactants. Surfactants reduce the external Laplace pressure, and, therefore, nano-sized nanobubbles can remain stable if an appropriate ratio of surfactants are in the shell. It is important to investigate the optimal ratio of surfactants and their role in the size and number of bubbles [2,27,28].

Most drug and gas delivery applications require the key characteristics of biocompatibility, biodegradability, ease of transport of molecules, cellular attachment, and drug loading abilities [29]. The composition of the shell plays an important role in determining the stiffness, echogenic response, permeability to diffusion, and renal clearance [2]. Phospholipids have an amphiphilic character, and they contain hydrophobic chains and hydrophilic headgroups, which give them a unique property of self-assembly around hydrophobic gas in the core in the presence of surfactants and emulsifiers. Owing to this self-assembly characteristic, phospholipid-shelled bubbles can be synthesized using a variety of techniques, including sonication, agitation, microfluidics, and emulsification. Acyl chains provide cohesiveness and stability to the phospholipids. The longer the acyl chains, the higher the stability of the bubbles. Various compositions have been employed by the researchers for synthesizing micro- and nanobubbles. The phase transition temperature is an important parameter for phospholipids because at this temperature the phospholipids undergo a transition from gel to liquid and their fluidity increases. The phase transition temperature also depends on the polar head group, the degree of saturation of hydrocarbon chains, and the acyl chain lengths. Nanobubbles are prepared above phase transition temperatures to achieve a homogenous composition, while they are stored below phase transition temperatures after preparation [7,30,31]. Phospholipids are biocompatible molecules, and various researchers have reported their non-toxicity. Nano-sized phospholipids tend to be more stable. Various lipid formulations have been reported by researchers for gas and drug delivery applications [3,32,33,34,35]. 

Polyethylene glycol (PEG) is a flexible, hydrophilic polymer, and it has been employed in various research studies as an emulsifier in the preparation of micro and nanobubbles and other biological applications [36,37,38,39,40,41,42]. PEGylation contributes considerably to imparting biocompatibility and enhancing the lifespan of nanobubbles. PEG has been incorporated into various compositions of lipids, polymers, and proteins to synthesize micro/nanobubbles and liposomes [39,40,43]. PEG derivatives can be utilized to incorporate functional groups into the shells of nanobubbles. The ratio of PEG components is critical for the preparation of nanobubbles, as it determines the sizes and characteristics of these particles.

The zeta potential plays an important role in the preparation of nanobubbles and liposomes as it determines the stability of particles, their cellular intake, and their resistance against coalescence. A cell membrane has a negative potential, and logically it might attract higher positively charged particles. However, higher negative particles with a zeta potential in the range of −45 mV have also been reported to exhibit a greater cellular uptake [31]. On the other hand, gene delivery applications utilize cationic lipids with a positive charge to form complexes with negatively charged genes [21,44].

Although ultrasound-mediated gas and drug delivery are gaining more attention, nanobubbles can be employed without employing ultrasound. Phospholipid shells are permeable to gas, and as the gas diffuses out as the bubble shrinks and eventually breaks owing to the increasing external Laplace pressure, thereby releasing the encapsulated drug [7]. 

Oxygen delivery through micro and nanobubbles is a key concept aimed at the reversal of hypoxia in tumor microenvironment gas and other drug delivery applications [27,28,34,45,46,47,48]. Nanobubbles can be utilized to create supersaturated fluids for oxygen delivery. In a separate study, we demonstrated the use of oxygen nanobubbles for the effective reversal of hypoxia [8]. We demonstrated that oxygen nanobubbles (ONBs) can be used for downregulation of the hypoxia-inducible factor-1α (HIF-1α) protein, which is of clinical significance in chemotherapy. Higher oxygen availability also favors the therapeutic efficacy of radiotherapy and photodynamic therapy [7] and, therefore, ONBs have many potential therapeutic applications. 

This study aims to identify the role of the composition and surfactants in the preparation of nanobubbles. To study this, we initially experimented with three different compositions to investigate the size, number of produced particles, and zeta potential of the nanobubbles. In further experiments, we selected one composition ratio and continued to evaluate its characterization, cytotoxicity, and ultrasound imaging. We were keen to investigate the role of the PEG surfactant on the size and zeta potential of oxygen nanobubbles. We found that ratios in the range of 90:10 and 80:20 are optimal, and, thus, we finally opted for 85:15.

## 2. Materials and Methods 

### 2.1. Materials

The phospholipids 1,2-distearoyl-*sn*-glycero-3-phosphocholine (DSPC), 1,2-distearoyl-*sn*-glycero-3-phosphoethanolamine-*N*-[amino(polyethylene glycol)-2000] (ammonium salt) (DSPE-PEG-2000-Amine), and 1,2-distearoyl-*sn*-glycero-3-phosphoethanolamine-*N*-[biotinyl(polyethylene glycol)-2000] (ammonium salt) (DSPE-PEG-2000-Biotin) were purchased from Avanti Polar Lipids (Merck, Kenilworth, NJ, USA), while Fluorescein-avidin conjugate (FITC-avidin) was purchased from Thermo Fisher Scientific ( Waltham, MA, USA). Dulbecco’s phosphate-buffered saline (DPBS) and chloroform were purchased from Sigma Aldrich (c St. Louis, MO, USA).

### 2.2. Preparation of Oxygen Nanobubbles

DSPC, DSPE-PEG-2000-Amine, and DSPE-PEG-2000-Biotin were dissolved in chloroform in molar ratios of 50:50:0, 80:20:0, 85:8:7, and 90:5:5. The chloroform was dried by heating the flask in a hot air oven at 80 °C, which resulted in a thin dried lipid layer using 10 mL DPBS. The total final concentration of lipids in all composition ratios was approximately 4.1 mg/mL. To dissolve these lipids in DPBS, a bath tub sonicator was heated to 60 °C, and sonication was performed until the entire lipid layer was dispersed in DPBS, forming a milky suspension. Next, the suspension was further sonicated with a tip-sonicator at 190 W for 5 min in the presence of a 99.9% oxygen supply via a cylinder to synthesize ONBs. Fluorescent bubbles were prepared by adding 100 µL of FITC-avidin to 10 mL suspension and centrifuging at 500 g for 10 min.

### 2.3. Characterization of Oxygen Nanobubbles

For the purpose of imaging, bright-field and fluorescence microscopy, transmission electron microscopy (TEM), and scanning electron microscopy (SEM) were employed. Micro-sized bubbles were collected for microscopic imaging, while SEM was performed for both micro- and nano-sized bubbles. TEM was employed for nano-size imaging. 

Samples for SEM were created by injecting microbubbles in 5% agarose gel. First, the agarose gel was heated to 100 °C. Then, ONBs were added during the cooling process when the temperature of the agarose gel was below 50 °C. The agarose gel was allowed to dry, and then thin layers of the dried gel were cut for imaging purposes. These layers were coated with Pt for SEM imaging. 

Samples for TEM were prepared by negative staining using uranyl acetate. First, a copper grid was dipped in nanobubbles and dried. Then, it was washed and negatively stained with 2% uranyl acetate solution. These grids were then utilized for TEM imaging. 

Dynamic light scattering (DLS) (Malvern, PA, USA) was also employed to analyze the size and zeta potential of nanobubbles. Samples were diluted at 1:100 for DLS reading. The zeta potential was measured using a disposable capillary cell (DTS1070). Nanoparticle tracking analysis (NTA) (Nanosight NS300, Malvern, PA, USA) was employed for counting number of particles/mL.

### 2.4. Cytotoxicity Tests

Cytotoxicity tests were conducted using NIH-3T3, MDA-MB-231 breast cancer cells, Chinese hamster ovary (CHO) cells, and human adipose derived stem cells (HADSCs). 1 × 10^4^ cells per well were seeded in a 96-well plate, and CCK-8 was used to determine cell viability after treatment was carried out with various concentrations of nanobubbles and lipid solution over a period of 24 h.

For a hemolysis test, different concentrations of ONBs (0.1, 5, 10, 100, and 200 μL/mL) and respective lipid constituents were added to 1 mL of sheep blood. The mixture was kept in an incubator for 24 h. Drakin’s solution was added in a 96-well plate, and the measurements were performed at 510 nm through a microplate reader. 10% Tryton-X100 was employed as a positive control.

### 2.5. Ultrasound Imaging

ONBs were inserted (dilution 1:10 in DPBS) in a balloon and compared with a tap water sample using a commercially available general electric (GE) ultrasound machine for three different transducers with frequencies of 3–5 MHz, 6–9 MHz and 6–15 MHz, respectively.

### 2.6. Statistical Analysis

GraphPad Prism software was used for statistical analysis and graphical representations of the data. T-tests were performed on the data, and the significance was checked. Non-significant values are indicated by ns in the results section, while the symbols “*, **, ***, and **** ” indicate *p*-values of less than 0.05, 0.01, 0.001, and 0.0001, respectively.

## 3. Results

Table 1 presents the compositions of samples in terms of the mass and molar ratio used during the experiments. We used DSPE-PEG-Biotin in two combinations to test the avidin/biotin interaction in the case of fluorescence ONBs. The total final concentration of lipids was kept at approximately 4.1 mg/mL by rehydrating the dried lipids in 10 mL DPBS. 

**Figure 1:**
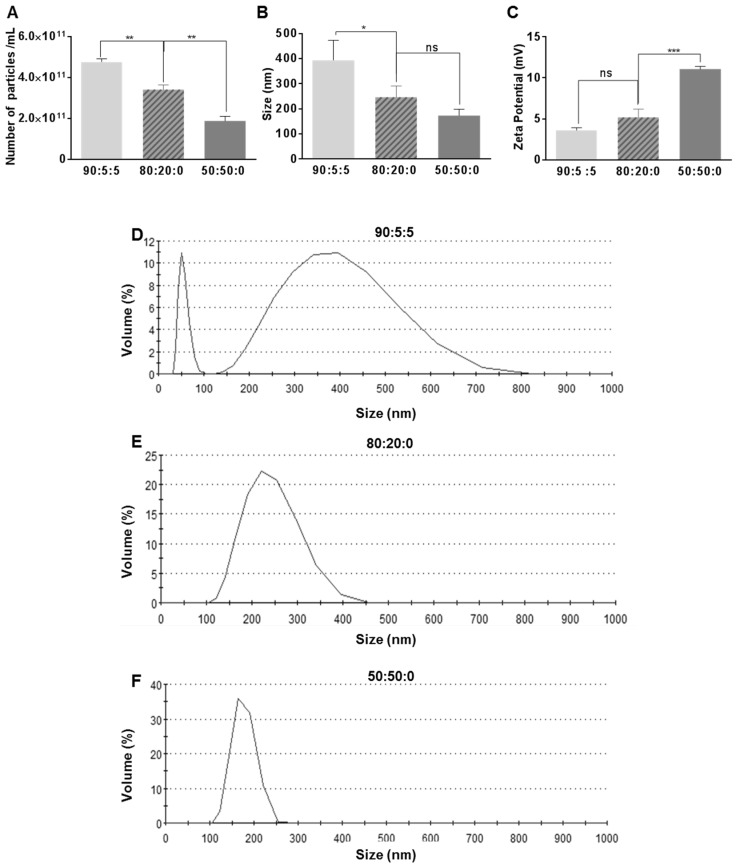
Impact of various compositions on the number (**A**), the size (**B**) and the zeta potential (**C**) of nanobubbles. Dynamic light scattering (DLS) data on the size distribution for 90:5:5 (**D**), for 80:20:0 (**E**), and for 50:50:0 **(F**). Here, *, **, ***, andindicate *p*-values of less than 0.05, 0.01, 0.001, and 0.0001, respectively.

Figure 1 illustrates the impact of the composition ratio on the size, the number of particles, zeta potential, and size distribution for the three composition ratios of 90:5:5, 80:20:0, and 50:50:0. Figure 1A shows that the number of particles decreases as we decrease the base lipid DSPC and increase the PEG component of the ONBs. In the case of 90:5:5, the number of particles is 4.77 × 10^11^ particles/mL, while in case of 80:20:0 it is 3.4 × 10^11^ particles/mL, which is 27% less. For the sample with a 50:50:0 composition, the number of particles is 1.88 × 10^11^ /mL, which is approximately 60% less than for the 90:5:5 sample. Figure 1B illustrates the impact of the composition ratio on the size. The ratio 90:5:5 exhibits a mean size of 394 ± 79 nm, 80:20:0 has a mean size of 246 ± 46 nm, and 50:50:0 has a mean size of 173 ± 25 nm. This reduction in size can be attributed to the high surfactant ratio. 

Figure 1C shows the increase in the zeta potential owing to an increase in the PEG ratio. The PEG surfactant contained an amine derivative, which might be responsible for the higher positive charge in the cases of 80:20:0 and 50:50:0. Figure 1D–F show the DLS data for the size distribution for the 90:5:5, 80:20:0, and 50:50:0 cases, respectively. The size distribution range for the 90:5:5 composition ratio exhibits two peaks: One at approximately 50 nm and another broad distribution from 150 nm to 750 nm with the peak containing approximately 10% of particles (Figure 1D). In the case of 80:20:0, the size range narrows to 100–400 nm and the peak contains approximately 25% of particles (Figure 1E). In the case of 50:50:0, the size range is from 100 nm to 250 nm (Figure 1F), which is significantly narrower than the two previous compositions, and the peak contains approximately 35% of particles, which is higher than the other two compositions. It can be observed that a higher PEG content leads to a narrower size distribution and decreased average size, as also indicated in Figure 1B.

**Figure 2:**
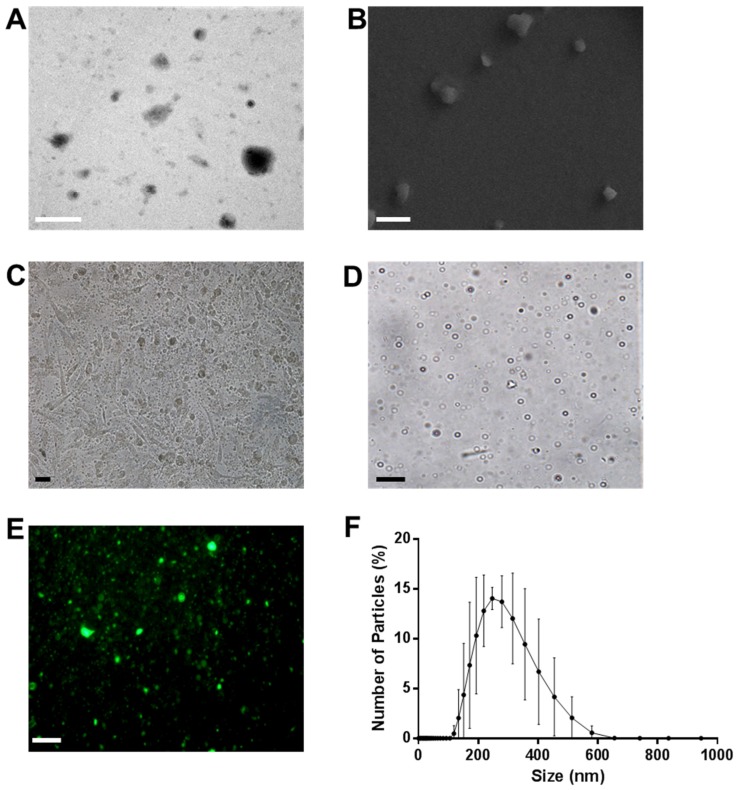
Characterization of oxygen nanobubbles (ONBs) with a lipid ratio of 85:8:7. (**A**) Transmission electron microscopy (TEM) image, with a scale bar of 500 nm. (**B**) Scanning electron microscopy (SEM) image, with a scale bar of 1 µm. (**C**) MDA-MB-231 cells with 10% ONBs in the media and a scale bar of 10 µm. (**D**) Micro-sized bubbles with a scale bar of 10 µm. (**E**) Fluorescent bubbles with a scale bar of 10 µm. (**F**) Combined DLS data of ONBs with *n* = 6.

Figure 2 illustrates the characterization of ONBs in the 85:8:7 composition ratio. Figure 2A shows a TEM image of ONBs. Figure 2B depicts an SEM image. These images contain both micro-sized and nano-sized particles. Figure 2C shows MDA-MB-231 cells injected with ONBs, captured through an optical microscope. Figure 2D shows an image of micro-sized particles on a glass slide in optical microscopy, while Figure 2E presents a fluorescent image of ONBs when FITC-avidin was conjugated with ONBs through an avidin-biotin interaction. Figure 2F depicts the size distribution of ONBs obtained through DLS. The mean size is approximately 300 nm, while the distribution range is mainly between 200 and 500 nm.

**Figure 3:**
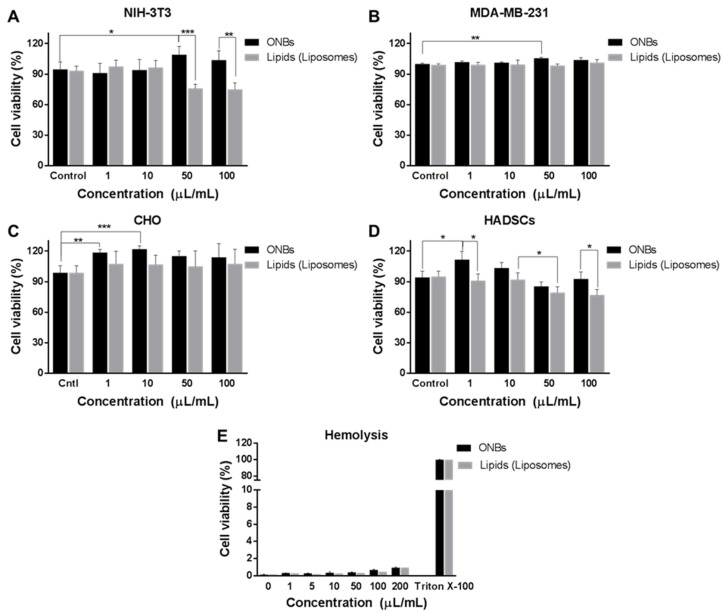
Cell viability test for ONBs determined through CCK-8. Cell viability of various concentrations of ONBs and lipids in NIH-3T3 (**A**) in MDA-MB-231 (**B**), in CHO (**C**) and in HADSCs (**D**) cell lines. (**E**) Hemolysis experiment for ONBs and lipids. Here, *, **, and *** highlight *p*-values of less than 0.05, 0.01, and 0.001, respectively.

Figure 3 illustrates the cell viability against various concentrations of ONBs and lipid constituents in various cell lines. Figure 3A shows the cell viability of ONBs and lipids in NIH-3T3 cells. The control ONB group has a cell viability of 94%, while 1 μL/mL and 10 μL/mL exhibit similar cell viabilities. However, in the 50 μL/mL case, cell viability is enhanced by 15% compared with that in the control case, and for 100 μL/mL, cell viability is enhanced by 7%. Conversely, the lipid constituents reduced cell viability by approximately 20% when they were utilized in 50 μL/mL and 100 μL/mL concentrations. This result indicates that a higher concentration of lipids reduces cell viability in NIH-3T3. However, ONBs in concentrations of 50 μL/mL and 100 μL/mL improved the cell viability by more than 30% as compared to lipids of similar concentrations, which can be attributed to the presence of oxygen and higher availability of oxygen. 

Figure 3B illustrates the cell viability in the case of MDA-MB-231 breast cancer cells. This indicates that the 1 μL/mL and 10 μL/mL concentrations exhibit similar cell viability as compared to the control group, while a concentration of 50 μL/mL enhances cell viability by 5%. In the case of MDA-MB-231, the lipid constituents do not exhibit any cytotoxicity, even at higher concentrations of 50 μL/mL and 100 μL/mL. When we compare this result with other cell lines, it is evident that tumor cells exhibit more tolerance to higher lipid concentrations. While 50 μL/mL ONBs exhibited an increase in cell viability, 100 μL/mL ONBs did not exhibit any significant difference during 24 h of the experiment. Therefore, 50 μL/mL proved to be more effective in this particular case.

Figure 3C shows the cell viability assay of CHO cells. In this case, 1 μL/mL and 10 μL/mL ONBs increased cell viability by approximately 20% compared with the ONB control, while concentrations of c increased cell viability by approximately 15%. The lipid constituents in this group did not cause a significant increase or decrease in cell viability compared with that in the control group. Higher concentrations of lipids, i.e., 50 μL/mL and 100 μL/mL, also did not exhibit any significant toxicity. 

Figure 3D shows the cell viability for HADSCs. Concentrations of 1 μL/mL and 10 μL/mL ONBs enhanced cell viability, while a concentration of 50 μL/mL reduced cell viability. In the case of lipids, 50 μL/mL and 100 μL/mL significantly reduced cell viability. The reason can be attributed to the lipid concentration. 

Figure 3E shows the result of the hemolysis experiment. The ONBs exhibited less than 2% hemolysis in all cases between 1 μL/mL and 200 μL/mL. In the case of lipid constituents, similar results were observed. From these results, we can observe that ONBs do not cause hemolysis.

**Figure 4:**
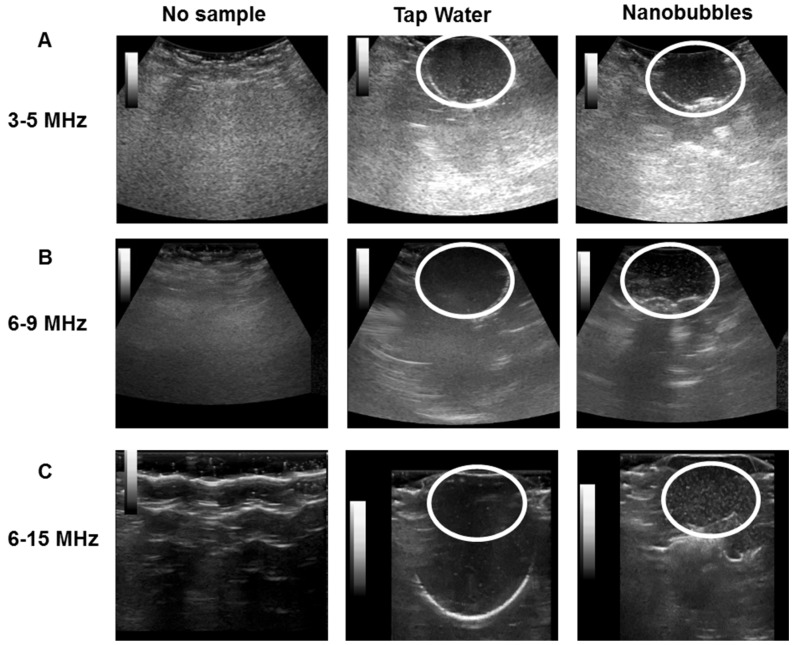
Ultrasound images of ONBs. No sample, tap water, and nanobubbles were compared. (**A**) shows images with a 3 to 5 MHz transducer. (**B**) shows images for a 6 to 9 MHz transducer. (**C**) shows images with a 6 to 15 MHz transducer.

Figure 4 illustrates the ultrasonic imaged of ONBs. Figure 4A show ultrasonic images captured with a 3 to 5 MHz curved transducer. We captured the screens with no sample, tap water, and nanobubbles, respectively. There is no visible difference between the tap water and nanobubbles. Since most micro size bubbles have a resonance frequency in the range of 3 to 5 MHz, these results suggest that our sample did not include many microsize particles to exhibit higher contrast as compared to tap water. It also indicates that nanobubbles do not generate significant echoes compared with tap water. Figure 4B show images acquired with a 6 to 9 MHz linear probe. It can be observed that the contrast provided by nanobubbles is slightly improved compared with tap water. Figure 4C present images captured with a 6 to 15 MHz transducer. The difference between the nanobubbles and tap water is visible. Thus, we can confirm that nanobubbles generate echoes at higher frequencies, and they can be traced using commercial ultrasound equipment. The mechanical index (MI) during these images was kept constant at 0.15. White circles in the figures indicate the area of a balloon containing ONBs. 

## 4. Discussion

In this study, we focused on investigating the effects of the shell composition on the size and characteristics of ONBs. We found that increasing the PEG ratio reduced the sizes of the bubbles and narrowed their distribution. The distribution range in the case of 90:5:5 was between 50 and 750 nm; this narrowed to 100 to 400 nm in the case of 80:20:0 and 100 to 250 nm in the case of 50:50:0. Reducing the size and narrowing down the size distribution is a desirable effect in nanobubble preparation because that way the enhanced permeability and retention (EPR) effect might be utilized for higher cellular uptake in theranostic applications. However, a higher PEG ratio also reduced the number of synthesized particles/mL during the process. The number of particles in the cases of 80:20:0 and 50:50:0 were reduced by 27% and 60%, respectively, compared with the 90:5:5 composition. Therefore, it can be concluded that a higher ratio of base phospholipid (>80%) is optimal for generating the higher number of particles. The zeta potential also considerably increased in the case of a 50:50:0 composition ratio compared with those of 90:5:5 and 80:20:0. Although higher zeta potential is favorable for some applications like gene delivery, other applications might prefer closer to neutral potential.

To optimize the composition ratio to obtain an optimal number of particles and narrow size distribution, we selected an 85:15 molar ratio containing 85% DSPC and 15% surfactant. The reason for this selection is that it imparts more functionality options by containing both amine and biotin functional groups, and we were able to synthesize fluorescent ONBs. By maintaining this composition ratio, we were also able to generate a higher number of particles in a narrower distribution. The 85:8:7 composition resulted in nanobubbles with a peak size of approximately 300 nm and distribution range of 200 to 400 nm, while the number of particles was 4.11 × 10^11^ particles/mL. This represents a tradeoff between a smaller size range and the number of generated bubbles. TEM and SEM imaging were conducted to observe the characteristics of ONBs. TEM image showed several ONBs in the size range of 100 nm or smaller. That might be due to the vacuuming effect during imaging. SEM image indicated the surface morphology, some of the particles were not spherical which might be the result of aggregation during sample preparation. Therefore, microsize particles were collected for optical microscopy and the bubbles are clearly observable with shell/core composition. The microscopic image of ONBs with MDA-MB-231 cells also indicate many ONBs in the cellular media. Biotin functional group was evaluated by synthesizing fluorescent ONBs using FITC-avidin.

Phospholipids are generally biocompatible, and PEG derivatives enhance the biocompatibility. PEGylation improves the stability and half-life of nanobubbles. It also imparts a resistance to coalescence. We tested the cell viability of ONBs in different cell lines, and it can be concluded that they are not cytotoxic for cells. The total concentration of phospholipids and PEG derivatives were kept approximately 4.1 mg/mL for all experiments and we compared the biocompatibility of ONBs with different lipid constituents (DSPC, DSPE-PEG-2000-amine, and DSPE-PEG-2000-biotin) to determine the role of oxygen in the biocompatibility and cytotoxicity. It is clearly observable that in most cases ONBs either improved or maintained cell viability. Even at higher concentrations, the cell viability in the case of ONBs was higher compared with the lipid counterparts. This clearly indicates that oxygen plays a positive role in enhancing cell viability. As indicated in the results for the cases of NIH-3T3 and AHDSCs, 50 μL/mL (5% *v*/*v*) and 100 μL/mL (10% *v*/*v*) ONBs exhibited higher cell viabilities compared with lipids of the same concentrations. Liposomes in these conditions reduced cell viability at 5% and 10% concentrations, which can be attributed to access amount of lipids damaging the cells. 

In the case of the breast cancer cell line MDA-MB-231, ONBs and lipids did not exhibit any cytotoxicity, while 5% ONBs exhibited an increase in cell viability as compared to the control group. This increase in cell viability may be attributed to a higher level of oxygen present which enhances cell differentiation. While 100 μL/mL ONBs did not increase cell viability, but they did not exhibit any cytotoxicity either in this case. A higher level of oxygen has been associated with downregulation of HIF-1α protein in tumors which is critical for tumor treatment. Further studies are required to investigate the optimal level of oxygen for tumor cells to enhance treatment. Tumor cells are an important target of drug delivery applications using nanobubbles, and our results exhibit that higher concentrations of ONBs and liposomes up to 10% of media volume can be employed in such cases for drug delivery. This is an important aspect as more drug can be loaded into the higher number of particles for anti-tumor drug delivery applications. 

In the case of CHO cell line, 1 µL /mL (0.1% *v*/*v*) and 10 μL/mL (1% *v*/*v*) ONBs were more beneficial for the enhancement of cell viability. Higher concentrations of ONBs and lipids, i.e., 50 μL/mL and 100 μL/mL did not cause a significant reduction in cell viability. All these results demonstrate that ONBs can be employed for delivering oxygen to cells and that they improve cell viability by enhancing the microenvironments of cells. 

Directly exposing blood to oxygen pressure may cause hemolysis, but ONBs do not cause hemolysis even at 20% (*v*/*v*), as shown by our results. This result is significant because hemolysis is a major issue when blood is directly exposed to oxygen pressure. In our study, ONBs released oxygen through a diffusion mechanism, which is a slow natural process, and, therefore, hemolysis did not occur. Therefore, it can be concluded that a higher level of oxygen (ONBs 10% of media volume) is generally not harmful to the cellular microenvironment and instead, it helps in improving the cell viability in most cases. Further, ONBs can be used to supply oxygen more than tissue partial pressure, and this method can be effectively used for reversal of hypoxic conditions in tumors as demonstrated previously by our group and other researchers. This aspect is of significant importance in anti-tumor applications related to chemotherapy, radiotherapy, and photodynamic therapy.

Ultrasonic imaging with commercial ultrasound machine revealed that these ONBs can be traced using ultrasound. Nanobubbles exhibited a higher contrast compared with tap water in ultrasound imaging at higher frequencies (6–15 MHz transducer), but this was not a very clear contrast difference, as is required for diagnostic applications. However, these bubbles can be traced using ultrasound, and, therefore, ultrasound can be employed for therapeutic purposes. At lower frequencies, in the range of 3 to 5 MHz, the contrast was not very significant compared with tap water. This is a further indication that our sample consisted of nano-sized particles and was generating a contrast at higher frequencies of ultrasound. This is more meaningful when nanobubbles are used for theranostic applications in combination with ultrasound.

## 5. Conclusions

Nanobubbles are a promising tool for diagnostic and therapeutic applications, and considerable research attention has been given to various applications of nanobubbles in medical fields. In this study, we summarized the role of the composition on the size and characteristics of oxygen nanobubbles, by employing different ratios of phospholipids and emulsifying PEG-derivatives. Increasing the PEG ratio was found to contribute to a reduction in size and distribution. However, a higher ratio of base lipids was required to generate a higher number of particles. The role of ONBs in the enhancement of cell viability was evaluated for various cell lines. Oxygen was supplied through ONBs, which were found to be non-toxic, and did not result in hemolysis in sheep blood. ONBs were employed in ultrasonic imaging, and it was observed that these ONBs can be traced using commercially available ultrasound transducers. Future studies are required to evaluate the impact of oxygen nanobubbles in tumor environment for longer time duration.

## Figures and Tables

**Table 1:** Composition ratios of phospholipid and surfactants.

Sample #	Combination	Mass (mg)	Molar Ratio
1	DSPC	29.53	90
	DSPE-PEG-2000-Amine	5.8	5
	DSPE-PEG-2000-Biotin	6.26	5
2	DSPC	22.02	80
	DSPE-PEG-2000-Amine	19.5	20
	DSPE-PEG-2000-Biotin	0	0
3	DSPC	9.08	50
	DSPE-PEG-2000-Amine	32.39	50
	DSPE-PEG-2000-Biotin	0	0
4	DSPC	25.2	85
	DSPE-PEG-2000-Amine	8.4	8
	DSPE-PEG-2000-Biotin	7.9	7
